# Training-Label Efficiency in Vision-Based Inspection of LNG Tank Cables: A Duplicate-Aware and Calibration-Controlled Benchmark of Supervised and Normal-Only Anomaly Detection

**DOI:** 10.3390/s26196237

**Published:** 2026-09-30

**Authors:** Youngju Cho, Jiyoon Hong, Heejun Park

**Affiliations:** Department of Industrial Engineering, Yonsei University, Seoul 03722, Republic of Korea; yjcho@yonsei.ac.kr (Y.C.); jy021023@naver.com (J.H.)

**Keywords:** industrial vision-based inspection, LNG tank, cable defect detection, label-efficient learning, anomaly detection, threshold calibration

## Abstract

Vision-based sensing is increasingly important for liquefied natural gas (LNG) tank manufacturing, where missed defects in cable-related components can affect monitoring, control, and safety functions. However, industrial image datasets often contain duplicate or near-duplicate imagery, operating thresholds can be optimistically tuned on held-out test data, and abnormal labels are costly to obtain. This study evaluates LNG tank cable defect detection under a duplicate-aware and calibration-controlled protocol that separates model training, threshold calibration, and held-out testing. Using the AI-Hub LNG tank quality inspection image dataset, we compare ResNet-18, EfficientNet-B0, and ConvNeXt-Tiny supervised classifiers; PatchCore, PaDiM, and STFPM normal-only training anomaly detectors; and ResNet-18 training-label-budget experiments that retain all normal training images while varying the number of labeled abnormal training images used for model fitting. Across 36 de-duplicated final runs, EfficientNet-B0 achieved the strongest fully supervised performance (AUROC 0.9999±0.0001, F1 0.9956±0.0029). PatchCore was the best normal-only training anomaly detector (AUROC 0.8909±0.0109, F1 0.8666±0.0228). In the percentage-budget experiment, 1% labeled abnormal training samples already exceeded PatchCore in AUROC, F1, and false-negative rate under the fixed labeled-calibration protocol; in a finer 4–64 abnormal-training-label sweep with 90 runs, the first training budget exceeding PatchCore across AUROC, F1, and false-negative rate with 16 labeled abnormal training images. These results quantify abnormal training-label efficiency under a fixed labeled-calibration protocol, rather than the total abnormal annotation burden required to deploy the complete operating classifier.

## 1. Introduction

Liquefied natural gas (LNG) carriers and LNG-fueled vessels depend on tanks and related subsystems that operate under cryogenic and safety-critical conditions. LNG leakage, structural degradation, and equipment failures remain major concerns in maritime and energy infrastructure because failures may propagate through operational, environmental, and human factors [1,2]. In this context, quality inspection is not only a production-efficiency problem but also part of a broader safety assurance process. Cable-related components are especially relevant because they support sensor power, monitoring signals, and control functions used by LNG tank systems and associated equipment. Additional LNG safety studies on bunkering risk assessment, high-pressure fuel-gas supply systems, LNG storage-tank stratification/rollover, and structural response under cryogenic and blast conditions further support the need for the preventive monitoring of LNG-related infrastructure rather than relying only on post-failure response [3,4,5,6].

Manual inspection is labor-intensive and can be inconsistent when defects are subtle, rare, or visually heterogeneous. Deep learning has therefore become a practical route for automated optical inspection in manufacturing and industrial quality control [7,8,9]. Fully supervised convolutional and transformer-based classifiers can perform very well when representative normal and abnormal labels are available [10,11,12]. However, supervised inspection has three limitations in industrial deployment. First, abnormal examples are usually scarce compared with normal samples. Second, annotation requires domain expertise and may be costly when defects are small or ambiguous. Third, high headline performance may be inflated when duplicate or near-duplicate images cross train/test boundaries or when operating thresholds are tuned on the test set [13]. Recent reviews of AI-based visual inspection and data-driven industrial fault diagnosis similarly identify data scarcity, model generalization, interpretability, and deployment constraints as persistent barriers to robust inspection systems [14,15,16].

Normal-only training anomaly detection offers a complementary paradigm. Instead of learning all possible abnormal classes, these models learn the distribution of normal images and flag deviations as anomalies. Embedding-based methods such as PatchCore, PaDiM, and student–teacher feature pyramid matching (STFPM) have become strong baselines for industrial anomaly detection because they use pretrained visual representations and do not require abnormal training labels [17,18,19,20,21]. Nevertheless, normal-only models can suffer from lower specificity, sensitivity, or threshold stability when the deployment domain contains high intra-class variation. For practitioners, the central question is therefore not simply which paradigm achieves the highest AUROC, but when the cost of abnormal labeling becomes justified.

This paper addresses that practical crossover question for LNG tank cable inspection. We study supervised classification, normal-only training anomaly detection, and abnormal training-label-budget supervised learning under a leakage-controlled and calibration-controlled protocol. Here, leakage control means that exact and near-duplicate groups are prevented from crossing train, calibration, and test splits; calibration control means that operating thresholds are selected only on a calibration split and then frozen before held-out test evaluation. The study is organized around four research questions:

1. RQ1. How well do supervised image classifiers perform for LNG tank cable defect detection under duplicate-aware and calibration-controlled evaluation?
2. RQ2. Which normal-only training anomaly detection model performs best, and how sensitive are its operating metrics to abnormal-label versus normal-only threshold calibration?
3. RQ3. How many labeled abnormal training images are required before supervised learning outperforms normal-only training anomaly detection under a fixed labeled-calibration protocol?
4. RQ4. What are the practical trade-offs among AUROC, F1, false-negative rate, and abnormal training-label budget for industrial inspection deployment?

The main contribution is an empirical benchmark showing, under a leakage-controlled and calibration-controlled protocol, how much abnormal training annotation is needed for supervised learning to outperform normal-only training anomaly detection in LNG tank cable inspection. Specifically, with all normal training samples retained, only 1% abnormal training labels was sufficient for a ResNet-18 classifier to exceed the best normal-only model, PatchCore, in AUROC, F1, and false-negative rate under a fixed labeled-calibration regime. A finer repeated label-count experiment further showed that a training set containing 16 labeled abnormal images was sufficient to exceed PatchCore across AUROC, F1, and false-negative rate under the fixed labeled-calibration protocol. This finding converts a general intuition—that supervised learning improves with labels—into a concrete training-label-efficiency result for this industrial inspection task, while avoiding the stronger and unsupported claim that 16 abnormal annotations are sufficient for the entire deployment pipeline.

## 2. Related Work

### 2.1. Visual Inspection for Industrial Defects

Machine-vision inspection has been widely studied for manufacturing quality assurance, including defective product inspection, automatic optical inspection, and surface-defect detection [7,8,9,22]. Earlier systems relied on handcrafted features and rule-based image processing, while modern systems increasingly use deep neural networks that learn defect-relevant features from data. Reviews of industrial surface-defect detection emphasize that deep learning can improve robustness but often requires careful dataset design, representative labels, and deployment-aware evaluation [9,22]. More recent surveys of AI-based industrial visual inspection and small-object detection further emphasize that subtle defects may lose discriminative features during feature extraction and that fair benchmarking must report not only accuracy but also efficiency and deployment constraints [14,23]. This is relevant for cable inspection because coating damage, scratches, and partial tearing may occupy only a small portion of the image. Studies of micro-defect detection in aerospace-grade metal surfaces provide a related example in which small cracks and point defects require localized visual evidence rather than only global image context [24].

For LNG and maritime infrastructure, visual inspection is part of a broader reliability and safety-management context. LNG leakage studies highlight the consequences of equipment failures and the need for monitoring systems that support early detection [1,2]. Although the present task is not gas-leak detection, the motivation is similar: missed defects in critical infrastructure can increase downstream risk.

### 2.2. Supervised Image Classification

Supervised convolutional networks remain strong baselines for binary defect classification. ResNet introduced residual learning to support deeper image classifiers [10]; EfficientNet introduced compound scaling to balance depth, width, and resolution [11]; and ConvNeXt modernized convolutional architectures using design choices inspired by vision transformers [12]. These models can achieve high accuracy when abnormal classes are well-represented in the training set. The practical drawback is annotation demand: collecting and labeling abnormal examples is expensive in industrial settings, and model performance may not transfer to unseen defect modes.

These supervised architectures were selected not as newly proposed algorithms, but as representative transfer-learning baselines with complementary design principles. ResNet-18 provides a compact residual CNN whose skip connections reduce optimization difficulty and make it a stable baseline for small industrial datasets [10]. EfficientNet-B0 tests whether compound scaling can improve the accuracy–efficiency trade-off for deployment-oriented inspection [11]. ConvNeXt-Tiny represents a modernized convolutional family and helps to determine whether more recent CNN design choices materially improve cable-defect classification beyond conventional residual and efficient networks [12]. Together, these models define the supervised performance ceiling under known-defect labeling.

### 2.3. Normal-Only Industrial Anomaly Detection

Normal-only anomaly detection trains with normal samples only and evaluates whether a test image deviates from the normal distribution. This setting is attractive for inspection because normal data are easier to collect and abnormal labels may be unavailable. PaDiM models patch-level embedding distributions with multivariate Gaussian statistics [17], PatchCore stores representative normal patch features and scores anomalies by nearest-neighbor distance in embedding space [18], and STFPM compares student and teacher feature pyramids to identify abnormal regions [19]. Surveys report that embedding-based models often outperform reconstruction-only autoencoders for industrial anomaly localization and detection [20,21]. Recent work has also extended this direction through outlier exposure, feature-consistency learning, and vision-language explanations for industrial anomaly inspection [25,26,27,28]. These studies are relevant as context, but they are not used as primary baselines here because the goal is to compare standard supervised and normal-only paradigms under a controlled label-budget protocol rather than to propose a new anomaly-detection architecture.

The three normal-only methods were chosen because they operationalize different mechanisms for detecting departures from normal cable appearance. PaDiM estimates local feature distributions from normal images and treats low-likelihood patches as anomalous, which is useful when defects appear as localized texture or shape deviations [17]. PatchCore constructs a memory bank of nominal patch embeddings and uses nearest-neighbor distance to identify image regions that are poorly represented by normal training examples; this design is often effective for high-recall industrial screening because it preserves local normal-pattern diversity while reducing memory through coreset sampling [18]. STFPM trains a student network to reproduce pretrained teacher features on normal images, so feature inconsistency at test time becomes an anomaly signal [19]. These mechanisms justify the comparison because LNG cable defects may manifest as localized damage, scratches, coating loss, or texture disruptions rather than global object-class changes.

### 2.4. Calibration, Duplicates, and Label Efficiency

Industrial benchmark results can be sensitive to evaluation design. If duplicate or near-duplicate images appear in both training and test sets, the measured generalization performance may be optimistic [13]. If the decision threshold is selected using test labels, the reported F1, sensitivity, specificity, and false-negative rate no longer reflect deployment conditions. We therefore separate model fitting, threshold calibration, and final testing. In addition, rather than comparing only the endpoints of no abnormal labels versus full abnormal supervision, we evaluate a controlled abnormal-label budget. This directly supports deployment planning by estimating the minimum annotation level needed before supervised learning becomes preferable.

This evaluation design is especially important for industrial anomaly detection because models can obtain high ranking metrics while still producing unsafe operating points after thresholding. Prior reviews emphasize that unsupervised industrial anomaly detection is strongly affected by defect scarcity, annotation cost, and domain-specific visual variability [20,21]. Therefore, the present benchmark treats calibration as part of the deployment protocol: AUROC and AUPRC measure score quality, whereas F1 and FNR evaluate the frozen operating threshold that an inspection system would actually use.

## 3. Materials and Methods

**Figure 1:**
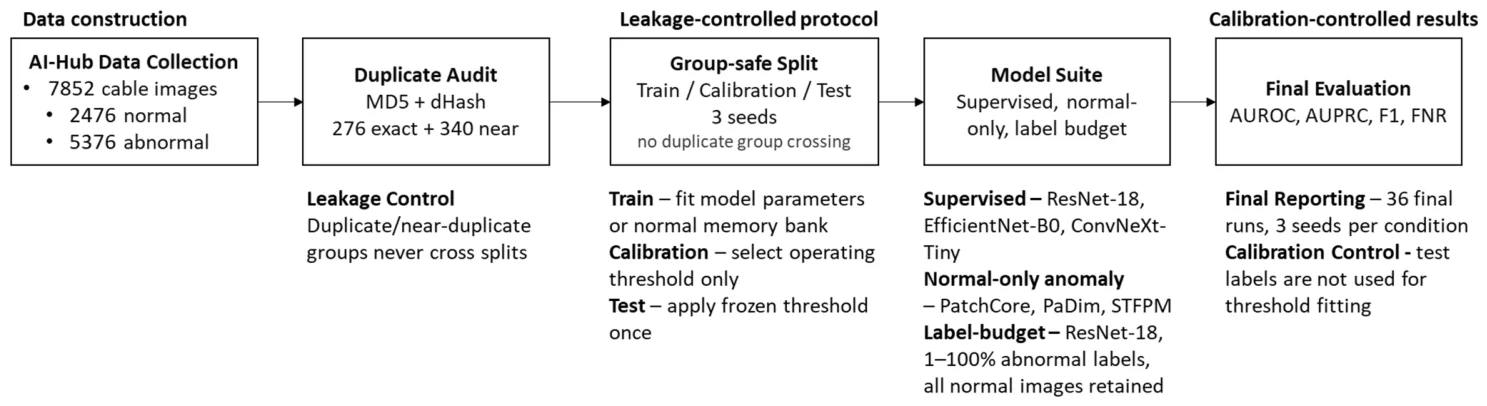
Research scheme. The AI-Hub LNG cable image subset was organized into a binary image-level dataset, audited for exact and near-duplicate images using MD5 and perceptual hashing, and partitioned into group-safe train, calibration, and test splits with no duplicate-group crossing. Supervised classifiers, normal-only anomaly detectors, and abnormal-label-budget supervised models were then evaluated using thresholds selected only on the calibration split and frozen before held-out test evaluation. AUROC, AUPRC, F1, and FNR are used as the primary reporting metrics.

The overall workflow of this study comprises data auditing, group-safe partitioning, model evaluation, and calibration-controlled testing, as illustrated in Figure 1.

### 3.1. Dataset

This study uses the cable subset of the AI-Hub LNG tank quality inspection image dataset [29]. According to the dataset release documentation, the dataset was constructed under the supervision of Mirae IT Co., Ltd. (lead organization) in collaboration with XyPlanet Co., Ltd., DSME Information Systems, Data Nuri Co., Ltd., and Geoje University Industry–Academic Cooperation Foundation, which participated in data-collection platform development, data processing, model development, and quality management. Because the public dataset metadata do not disclose the specific camera hardware, optical sensor models, manufacturer name, or manufacturing location, original equipment sourcing details cannot be reported.

**Figure 2:**
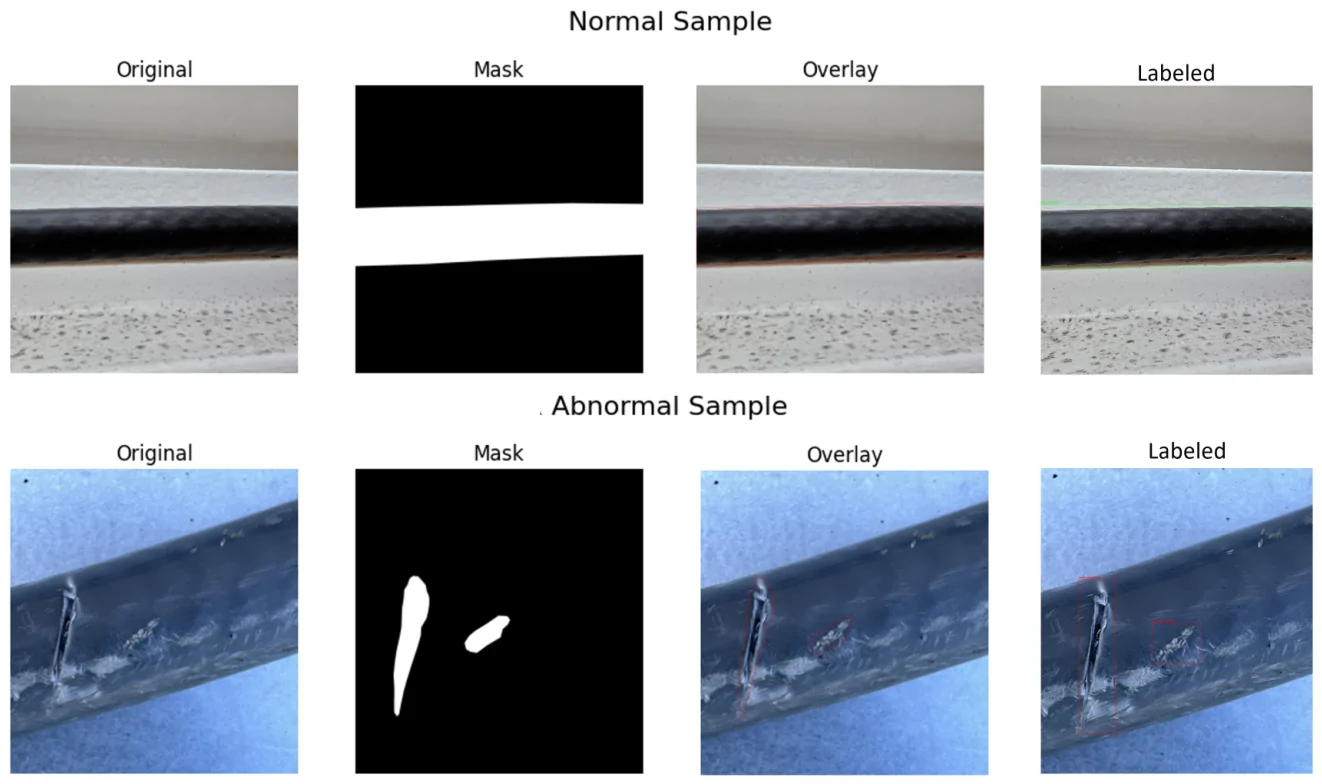
Representative LNG tank cable inspection images. The **upper row** shows a normal cable image, and the **lower row** shows an abnormal cable-damage case with annotation mask and labeled defect region. These examples illustrate the image-level binary inspection task used in this study; localization masks are shown only to visualize defect appearance and were not used as the primary evaluation endpoint.

The original extracted cable subset contained 7852 images: 2476 normal images and 5376 abnormal images. The original source split contained 2175 normal and 4785 abnormal training images, and 301 normal and 591 abnormal validation images. The abnormal class was dominated by cable damage, with rare co-defect labels recovered from JSON metadata. The experiment used image-level binary labels: normal cable versus abnormal cable as shown in Figure 2. The dataset preparation workflow began by extracting only the cable-related image and label archives from the larger LNG tank inspection dataset. Directory names and JSON metadata were harmonized into a binary manifest containing image path, original source split, class name, numerical label, source archive, hash values, and recovered defect-type fields. The recovered abnormal metadata indicated one dominant primary defect, cable damage, plus rare co-defect combinations involving coating detachment or scratches. These rare co-defects were retained in the binary abnormal class rather than discarded, because the intended deployment decision is whether an image should be escalated for inspection.

### 3.2. Duplicate-Aware and Calibration-Controlled Protocol

The evaluation protocol was designed to reduce two common sources of optimistic bias identified in machine-learning evaluation: leakage between model-development and final-test data, and operating-point selection that is not separated from the final evaluation [13]. First, exact duplicates and near-duplicates were audited using file hashes and perceptual hashes. The audit found 276 exact-duplicate rows and 340 near-duplicate rows by difference hash. Conservative duplicate groups were constructed so that duplicate or near-duplicate images could not cross train, calibration, and test splits. Across the final protocol splits, the duplicate/near-duplicate group crossing count was zero.

Second, the original training and validation partitions were recombined and repartitioned into train, calibration, and held-out test splits using group-aware splitting. The selected split proportions were approximately 60% train, 20% calibration, and 20% test, with class prevalence preserved across splits. Table 1 summarizes the split sizes for the three seeds used in the final reporting. The calibration split was used only to choose the operating threshold, and the threshold was then frozen before evaluating the held-out test split. Unless otherwise stated, thresholds were selected by Youden’s index on the calibration scores.

Duplicate groups were defined conservatively. If an image belonged to an exact duplicate set by MD5 hash, that exact-hash group was used. Otherwise, if it belonged to a repeated perceptual-hash group, the perceptual-hash group was used. Images without repeated exact or perceptual hashes were assigned unique groups. Candidate train/calibration/test partitions were sampled with group-aware splitting and then scored for class-balance and defect-coverage stability; the selected split minimized the candidate penalty while enforcing that no group appeared in more than one split.

### 3.3. Models

Three supervised image classifiers were evaluated: ResNet-18, EfficientNet-B0, and ConvNeXt-Tiny. All were trained as binary classifiers with ImageNet-style preprocessing at 224×224 resolution. Each model was trained for five epochs per seed.

Three normal-only training anomaly detection models were evaluated with a ResNet-18 backbone: PatchCore, PaDiM, and STFPM. These models were fitted only with normal images from the training split. The primary calibrated setting used both normal and abnormal calibration images for operating-threshold selection and then evaluated the frozen threshold on the held-out test split. To avoid conflating normal-only model fitting with fully annotation-free deployment, additional normal-only threshold variants were generated using only normal calibration scores, including the 95th and 99th percentiles of normal anomaly scores.

To quantify training-label efficiency, we also evaluated ResNet-18 under an abnormal training-label-budget protocol. All normal training images were retained, while abnormal training images were sampled at ratios of 1%, 5%, 10%, 20%, 50%, and 100%. With the final splits, the 1% setting corresponds to approximately 33 labeled abnormal training images per seed, while the 5%, 10%, 20%, 50%, and 100% settings correspond to approximately 162, 323, 646, 1615, and 3229 abnormal training labels, respectively. The labeled calibration split was held fixed across budgets and contained both normal and abnormal images, including approximately 1070 abnormal calibration images per seed. Therefore, the label-budget experiments quantify abnormal training-label efficiency under a fixed labeled-calibration regime rather than the total annotation burden required to construct an operating classifier. The calibration abnormal labels are held constant across budgets and are not included in the reported training-label counts.

The role of each model family in the benchmark is therefore distinct. The fully supervised classifiers estimate the best achievable performance when defect labels are available; the normal-only training anomaly detectors estimate cold-start model fitting without abnormal training labels; the normal-only threshold variants estimate a more restrictive deployment rule without abnormal calibration labels; and the label-budget ResNet-18 experiments estimate the transition point where limited abnormal training annotation becomes more valuable than normal-only modeling under the same labeled-calibration protocol. This design allows the study to answer not only which algorithm performs best, but also which learning paradigm is appropriate at different abnormal training-label budgets.

### 3.4. Training and Implementation Details

All data preprocessing, model training, and evaluation pipelines were executed in Google Colaboratory (Google LLC, Mountain View, CA, USA; https://colab.research.google.com/, accessed on 20 May 2026). The computational workflow was implemented in Python (v3.12.0) using PyTorch (v2.3.0), torchvision (v0.18.0), Anomalib (v2.4.1), scikit-learn (v1.4.2), NumPy (v1.26.4), pandas (v2.2.2), and Matplotlib (v3.8.4). All dependency versions and random seed states were frozen in an execution environment manifest to guarantee deterministic pipeline execution and experimental reproducibility. All supervised classifiers were initialized with torchvision pretrained weights and modified with a two-class output layer. Images were resized to 224×224 and processed with ImageNet-compatible normalization. Models were trained with cross-entropy loss, AdamW optimization, learning rate $1\times 10^{-4}$, weight decay $1\times 10^{-4}$, batch size 32, and five epochs. For each epoch, training loss and elapsed time were saved; the checkpoint with the lowest training loss was retained before calibration and test scoring.

Normal-only anomaly models were implemented through Anomalib using the Folder datamodule. The protocol split was materialized into an Anomalib-compatible view with train/good, calibration/good, calibration/abnormal, test/good, and test/abnormal directories. PatchCore used ResNet-18 layer2 features with coreset sampling ratio 0.005 and nearest-neighbor scoring. PaDiM used ResNet-18 layers 1–3, and STFPM used ResNet-18 layers 1–2. Anomalib models were trained with batch size 1 and evaluated separately on calibration and test views so that the fitted memory bank or teacher–student representation was not refit during test evaluation.

For all three anomaly detectors, the output used in this study is an image-level anomaly score derived from local feature evidence. PatchCore scores an image by the distance between test patch embeddings and the nearest normal-memory features, PaDiM aggregates patch-level likelihood deviations from learned normal feature distributions, and STFPM aggregates teacher–student feature discrepancies. These image-level scores are well-aligned with the inspection decision studied here: whether a cable image should be accepted as normal or escalated as abnormal.

The label-budget experiment used the same ResNet-18 training procedure as the fully supervised baseline, but changed only the number of labeled abnormal samples used for model fitting. All normal training samples were always retained. For each seed and budget, the abnormal subset was sampled from the seed-specific train split, the model was trained from pretrained initialization, calibration scores were generated on the fixed calibration split, and the frozen threshold was applied to the held-out test split. The labeled calibration set is therefore not counted as part of the abnormal training-label budget; consequently, threshold-dependent metrics such as F1 and FNR reflect performance under a fixed labeled-calibration protocol.

The experiments were conducted in Google Colaboratory using PyTorch and Anomalib (v2.4.1). The notebook environment snapshot recorded Python (v3.12.13), PyTorch (v2.11.0+cu128), CUDA 12.8, and an NVIDIA Tesla T4 GPU (NVIDIA Corporation, Santa Clara, CA, USA). All final conditions used seeds 11, 22, and 33.

### 3.5. Grad-CAM Shortcut Sanity Check

To assess whether the best supervised model relied primarily on cable-region evidence rather than obvious background shortcuts, we generated qualitative Grad-CAM visualizations for the best-performing supervised run. The selected run was the EfficientNet-B0 classifier, and the target layer was the final feature block used by the PyTorch implementation. For every displayed image, Grad-CAM was computed with respect to the abnormal output class, including true-negative images, so that the heat map shows the regions that would increase the defect score. Heat maps were min–max-normalized by the Grad-CAM implementation, resized to the model input resolution, and overlaid on the RGB inspection image. Examples were selected from the held-out prediction file by confusion-matrix category, with up to three true-positive, true-negative, false-positive, and false-negative cases, when available. These visualizations are used only as a qualitative shortcut sanity check and are not reported as pixel-level localization metrics.

### 3.6. Metrics and Statistical Reporting

All final results are reported as mean ± standard deviation across three seeds (11, 22, and 33). The primary threshold-independent metric is AUROC. Since the dataset is imbalanced toward abnormal images, AUPRC is also reported, consistent with industrial anomaly-detection practice, where ranking and precision–recall behavior are often evaluated together [20,21]. Threshold-dependent metrics include F1, false-negative rate (FNR), sensitivity, specificity, accuracy, and Matthews correlation coefficient (MCC). FNR is emphasized because missed defects are particularly costly in industrial inspection and safety-critical LNG monitoring contexts [1,2,4].

For every main run, calibration scores were converted into a binary operating threshold before test evaluation. With Youden calibration, the selected threshold maximized sensitivity plus specificity minus one on the calibration split [30]. That threshold was written to a run-level protocol artifact and reused without modification on the held-out test split. Thus, AUROC and AUPRC measure ranking quality, whereas F1, FNR, specificity, accuracy, and MCC measure a frozen operating point. For anomaly detectors, we explicitly distinguish normal-only training with labeled calibration from stricter normal-only thresholding based only on normal calibration scores. For the supervised label-budget experiments, threshold-dependent metrics should be interpreted under this fixed labeled-calibration protocol; the training-label budget varies only the abnormal labels used for model fitting.

The final manuscript index contained 57 candidate main runs before duplicate-condition pruning and 36 final runs after deduplication. Each final main condition had three complete seeds and complete artifacts, including calibration predictions, test predictions, threshold metadata, and metrics. Additional sensor-revision analyses included 18 normal-only threshold variants and 90 fine label-count crossover runs.

## 4. Results

### 4.1. RQ1: Supervised and Normal-Only Training Benchmarks

Table 2 reports the main comparison between supervised and normal-only training anomaly-detection models. Even under duplicate-aware splitting and calibration-only threshold selection, supervised models achieved near-perfect discrimination. EfficientNet-B0 was the strongest fully supervised model, with AUROC 0.9999±0.0001, AUPRC 1.0000±0.0000, F1 0.9956±0.0029, and FNR 0.0072±0.0044. ConvNeXt-Tiny and ResNet-18 were also highly accurate, with AUROC values above 0.999 and F1 values above 0.993.

These results answer RQ1 by showing that supervised classifiers remain the performance ceiling for this dataset when abnormal labels are available. Importantly, the result is not produced by test-threshold tuning: each operating threshold was calibrated before held-out test evaluation.

Algorithmically, this behavior is expected when the abnormal category is represented in training: supervised CNNs directly optimize class separation between normal and defective cables, whereas normal-only training methods must infer abnormality indirectly as a deviation from normal appearance. Thus, the supervised models provide the appropriate upper-bound comparator for the label-efficiency analysis.

### 4.2. RQ2: Best Normal-Only Training Anomaly Detector

Among normal-only training models, PatchCore-ResNet18 performed best overall. It achieved AUROC 0.8909±0.0109, AUPRC 0.9373±0.0117, F1 0.8666±0.0228, and FNR 0.1519±0.0653. PaDiM ranked second in AUROC and F1, while STFPM showed the highest FNR and lowest AUROC among the three anomaly detectors.

**Figure 3:**
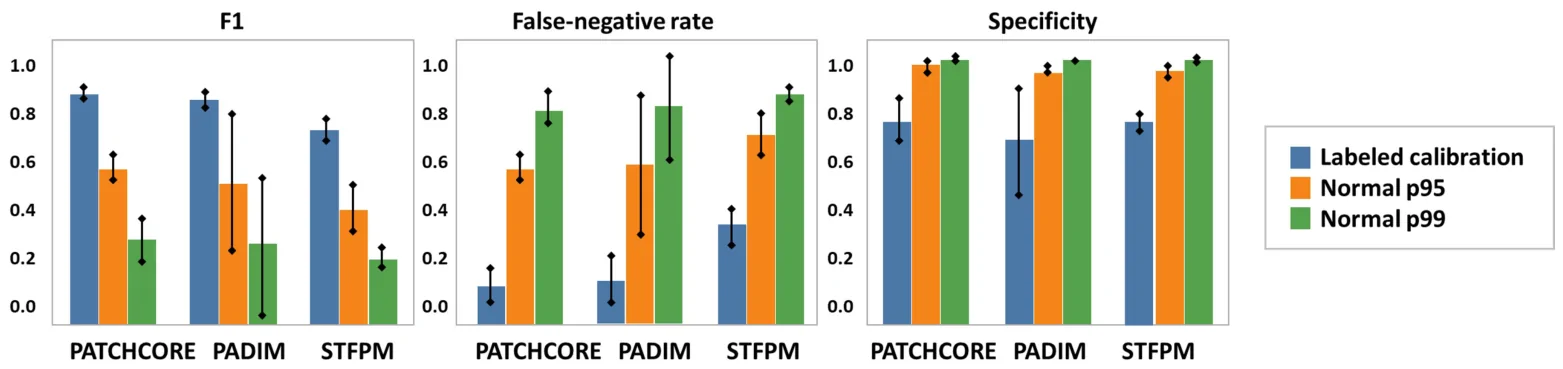
Normal-only threshold sensitivity analysis comparing labeled calibration with p95 and p99 normal-only thresholds. Bars show run means, and vertical error bars with diamond-shaped caps show standard deviations. The p95 and p99 thresholds were selected only from normal calibration anomaly scores.

This result indicates that embedding-memory approaches are useful when abnormal labels are not available for model fitting, but their operating performance remains meaningfully below supervised learning in this LNG cable setting. Because the main calibrated anomaly setting uses labeled abnormal calibration samples for threshold selection, the comparison should be interpreted as normal-only training rather than a completely annotation-free deployment regime. Figure 3 shows that the threshold rule materially changes the operating behavior of these models. For PatchCore, labeled calibration produced F1 0.8666±0.0228, FNR 0.1519±0.0653, and specificity 0.7660±0.0732. In contrast, the normal-only 95th- and 99th-percentile rules visually increased specificity to approximately 0.96 and 0.99, respectively, but also increased the false-negative rate to roughly 0.58 and 0.81 and reduced F1. Thus, without abnormal calibration labels, normal-only thresholding becomes much more conservative about raising alarms: false positives decrease, but missed defects increase substantially. The 95th and 99th percentiles were selected as simple pre-specified industrial operating rules because they correspond to allowing approximately 5% or 1% of normal calibration images to exceed the alarm threshold, respectively, without inspecting abnormal labels.

### 4.3. RQ3: Abnormal Training-Label Budget Needed to Surpass Normal-Only Detection

**Figure 4:**
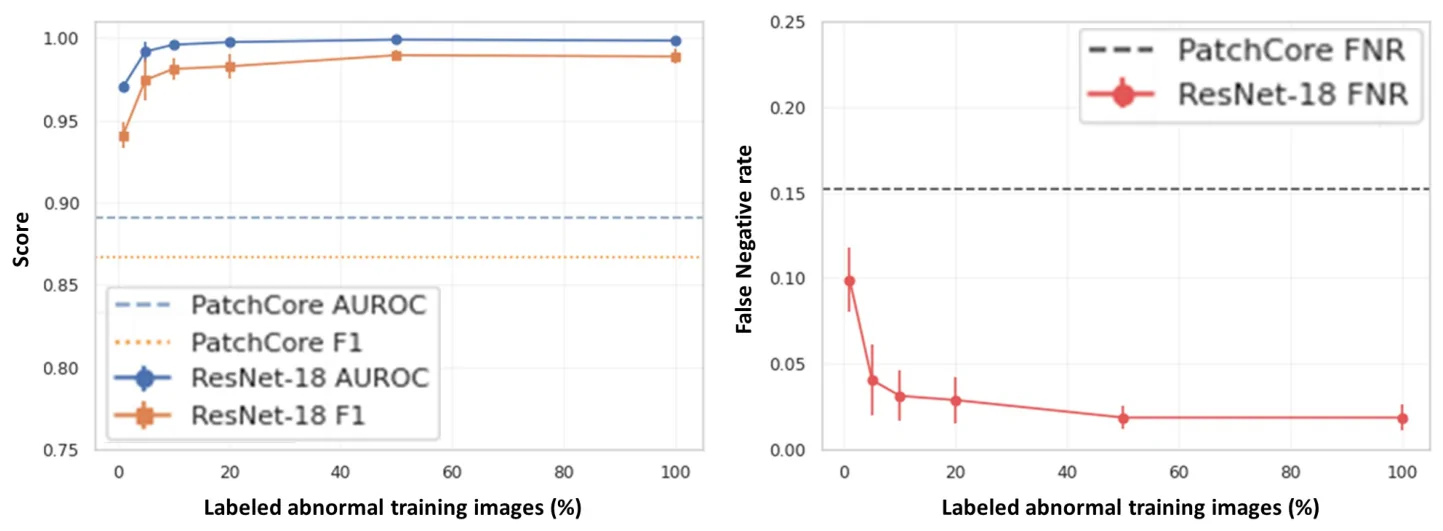
Percentage abnormal training-label budget curve for ResNet-18 compared with PatchCore. Circles show AUROC or FNR, squares show F1, and horizontal reference lines show PatchCore.

Table 3 reports the abnormal training-label-budget experiment, and the corresponding performance trends across budgets are illustrated in Figure 4. The strongest normal-only training model, PatchCore, provides the comparison point: AUROC 0.8909±0.0109, F1 0.8666±0.0228, and FNR 0.1519±0.0653. With all normal training images retained, ResNet-18 exceeded this normal-only benchmark using only 1% of the abnormal training labels for model fitting. At this training budget, ResNet-18 achieved AUROC 0.9702±0.0036, F1 0.9408±0.0076, and FNR 0.0990±0.0185.

**Figure 5:**
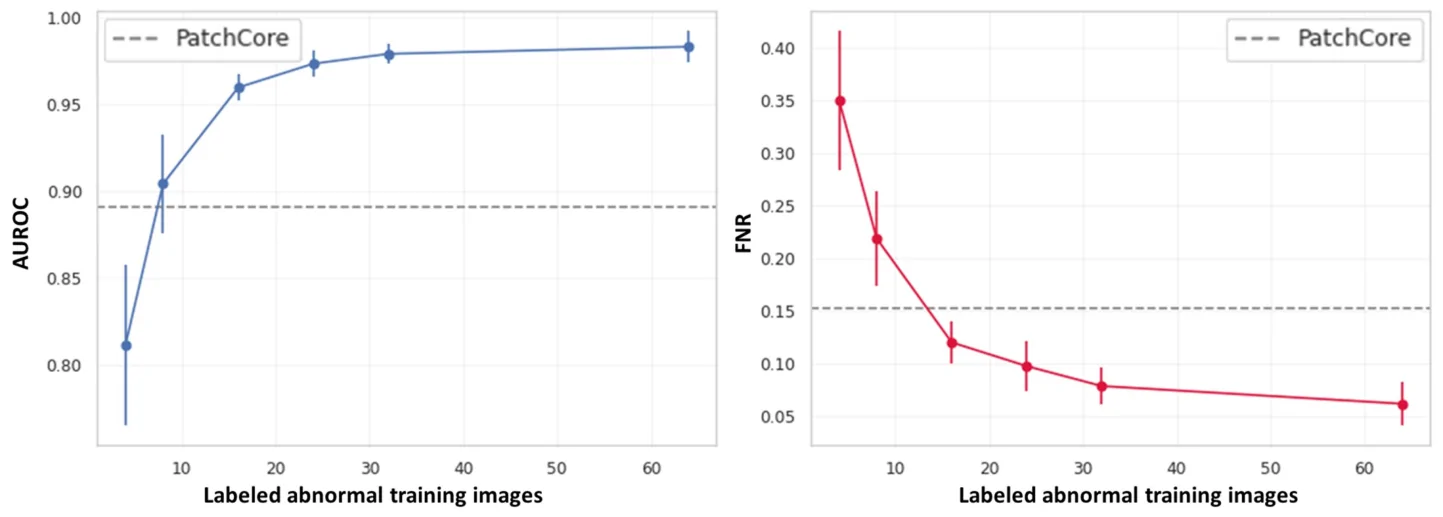
Fine abnormal training-label count crossover analysis. Circles show AUROC or FNR, squares show F1, and horizontal reference lines show PatchCore.

The percentage-budget experiment therefore shows that supervised learning exceeded PatchCore from the smallest evaluated percentage budget of 1%, corresponding to approximately 33 labeled abnormal training images. To refine this result, we additionally ran a finer label-count sweep using 4, 8, 16, 24, 32, and 64 labeled abnormal images, with three seeds and five subset draws per count. The quantitative outcomes are detailed in Table 4, and the corresponding crossover trajectories for AUROC and FNR are shown in Figure 5.

The fine-count results sharpen the annotation-budget interpretation. With only eight labeled abnormal training images, ResNet-18 exceeded PatchCore in AUROC but not in F1 or FNR. With 16 labeled abnormal training images, ResNet-18 exceeded PatchCore across AUROC, F1, and FNR. Thus, under this dataset and fixed labeled-calibration protocol, the observed practical training-label crossover occurs between 8 and 16 labeled abnormal training images for the combined ranking and operating-point criteria. This value should be interpreted as an observed dataset-specific crossover, not as a universal annotation requirement for all industrial cable-inspection datasets.

### 4.4. RQ4: Deployment Trade-Offs

The trade-offs are clearest when AUROC, F1, FNR, abnormal training-label budget, and calibration requirements are considered together. Fully supervised EfficientNet-B0 produced the best overall detection quality, but requires full abnormal annotation. PatchCore requires no abnormal training labels for model fitting and achieved the best normal-only training performance, making it a reasonable cold-start option. However, its calibrated FNR of 0.1519±0.0653 means that a meaningful fraction of abnormal test images would be missed at the selected operating point. The 1% label-budget ResNet-18 reduced the FNR to 0.0990±0.0185, the 5% budget reduced it further to 0.0405±0.0208, and the fine-count sweep showed that a training set containing 16 labeled abnormal images was sufficient to improve FNR relative to PatchCore under the fixed labeled-calibration protocol.

For industrial inspection deployment, these results suggest a staged strategy. When no abnormal labels are available for model fitting, PatchCore can be deployed as a normal-only training baseline or triage model, preferably with a pre-specified normal-only threshold rule if abnormal calibration labels are also unavailable. Once a small labeled abnormal training set is available, supervised learning becomes preferable under the fixed labeled-calibration setting evaluated here. If missed defects are the dominant operational risk, the 5% abnormal training-label budget offers a much stronger FNR reduction than the 1% budget while still requiring only a small fraction of abnormal training labels. If annotation resources are less constrained, fully supervised EfficientNet-B0 provides the strongest calibrated performance.

## 5. Discussion

The benchmark supports four main observations. First, leakage control and calibration control did not eliminate the superiority of supervised classifiers. Even after preventing duplicate-group crossings and freezing thresholds selected on calibration data, EfficientNet-B0, ConvNeXt-Tiny, and ResNet-18 all achieved near-perfect AUROC and F1. This strengthens the conclusion that labeled cable defects in this dataset are visually learnable by standard image classifiers.

Second, PatchCore was the best normal-only training model in the final protocol. This differs from the older draft result, where PaDiM appeared strongest, and illustrates why the final protocol matters. After final deduplication, three-seed reporting, and calibration-controlled run selection, PatchCore had the strongest AUROC, F1, and FNR among the normal-only training methods.

This result is consistent with the algorithmic role of PatchCore. As PatchCore compares test patches against a memory bank of normal patch embeddings, it can preserve multiple normal cable appearances and identify localized deviations without requiring an explicit abnormal class. In contrast, PaDiM compresses normality into parametric patch distributions, and STFPM relies on student–teacher feature consistency; these mechanisms can be effective but may be more sensitive to normal-image variability and calibration threshold choice in this cable dataset.

Third, the abnormal training-label-budget result is the central practical contribution. Normal-only training anomaly detection is attractive because it avoids abnormal labels during model fitting, but in this task it is overtaken quickly once abnormal labels are introduced. The 1% abnormal training-label setting already exceeded PatchCore, and the fine-count sweep refined the practical training-label transition to between 8 and 16 labeled abnormal training images when AUROC, F1, and FNR are considered together under a fixed labeled-calibration regime. For deployment teams, this means that a modest abnormal training-label campaign may be more valuable than investing only in increasingly complex normal-only models.

**Figure 6:**
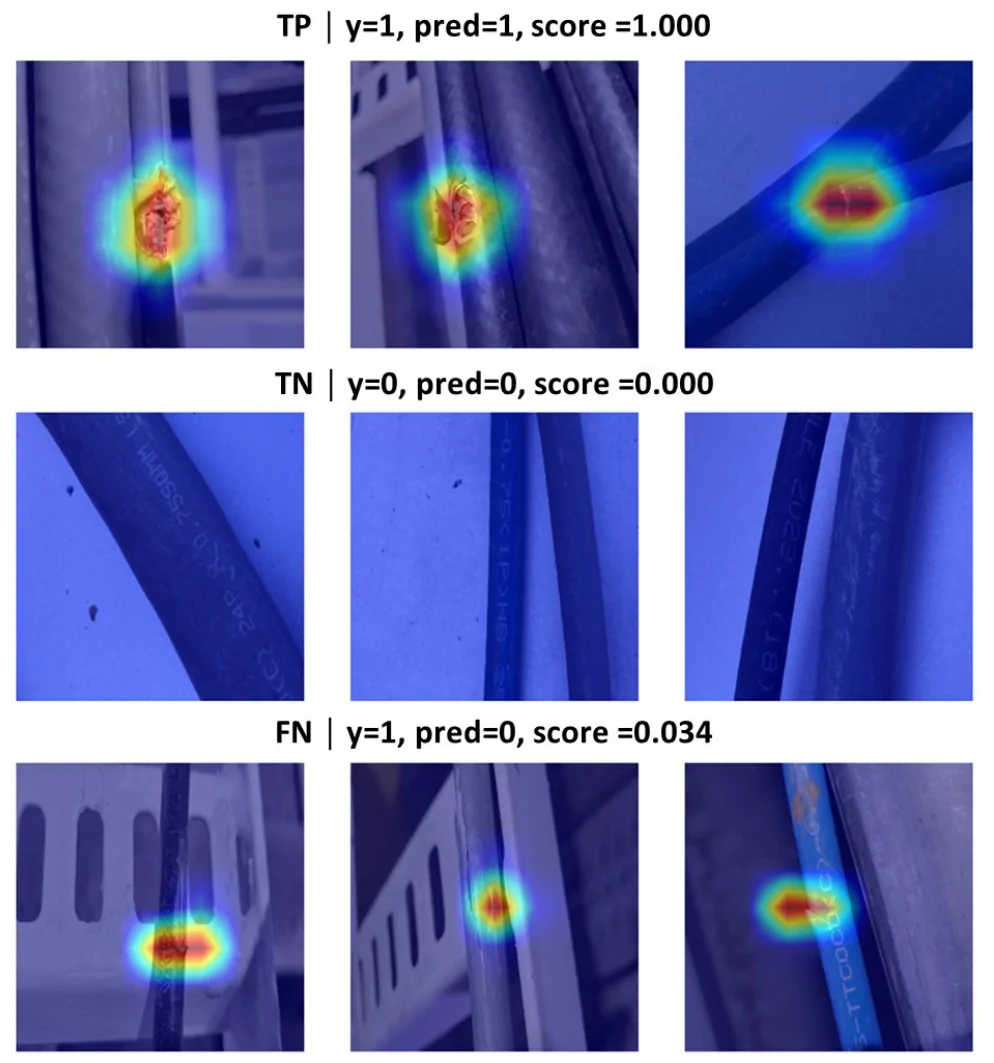
Grad-CAM shortcut sanity check for the best supervised model (EfficientNet-B0). Heat maps depict class-activation intensities with respect to the abnormal defect class: warm colors (red and yellow) denote regions of high model attention contributing positively to defect prediction, whereas cool colors (blue and cyan) indicate low or negligible relevance.

Fourth, metric choice affects the recommended operating point. AUROC summarizes ranking quality but does not specify how many defects will be missed after thresholding. F1 balances precision and recall but may still obscure safety priorities. FNR directly reflects missed abnormal images and is therefore especially important for inspection. In this benchmark, the 5% training-label-budget model provides a meaningful compromise: it greatly reduces missed defects relative to PatchCore while requiring far fewer abnormal training labels than full supervision under the fixed labeled-calibration protocol. To evaluate whether predictions were driven by genuine defect cues rather than background artifacts, qualitative Grad-CAM visualizations were generated for the top-performing supervised model, as illustrated in Figure 6. These visualizations serve strictly as a qualitative sanity check rather than a quantitative localization evaluation. In the displayed examples, two of three true-positive cases showed substantial activation inside the annotated defect region (attention ratio approximately 0.36), whereas true-negative examples had near-zero activation under the same abnormal-class target; one false-negative case showed only weak localized activation (approximately 0.10).

### Limitations

This study focuses on the cable subset of a public LNG tank quality inspection dataset. The results may not generalize to other LNG tank components, such as welds, insulation, membranes, or painted surfaces, where defect morphology and background variation differ. The dataset is also dominated by cable damage, with rare co-defect categories. Therefore, the label-budget crossover may be more favorable to supervised learning than it would be in a setting with many visually distinct abnormal modes, and the observed 8–16 abnormal-training-label transition should not be interpreted as a universal property of industrial inspection.

The benchmark is image-level. Localization outputs, pixel-level masks, and Grad-CAM visualizations were explored in the notebook workflow, but pixel-level localization is not claimed as a primary result of this manuscript. Since all normal training images are retained while the number of abnormal training images increases, the training-label curve reflects the combined effect of more abnormal examples, greater abnormal diversity, and a changing training-class ratio; it does not isolate sample count, class balance, and defect-space coverage as separate causal factors. Future work should extend the duplicate-aware and calibration-controlled protocol to pixel-level localization metrics, prospective production data, and unknown-defect holdout designs with sufficient non-base defect examples, as recent industrial anomaly-detection reviews emphasize both image-level and localization-oriented evaluation across 2D and 3D industrial settings [20,31,32]. A further extension would be to evaluate outlier-exposure methods and vision-language anomaly detectors under the same protocol, because these methods may improve interpretability or reduce threshold dependence but could also require auxiliary abnormal data or prompt-design choices that change the annotation-budget comparison [25,27,28].

## 6. Conclusions

This study benchmarked supervised classifiers and normal-only training anomaly-detection models for LNG tank cable defect detection under duplicate-aware and calibration-controlled evaluation. Supervised classifiers achieved the highest final performance, with EfficientNet-B0 reaching AUROC 0.9999±0.0001, F1 0.9956±0.0029, and FNR 0.0072±0.0044. PatchCore was the best normal-only training anomaly detector, with AUROC 0.8909±0.0109, F1 0.8666±0.0228, and FNR 0.1519±0.0653.

The key deployment finding is that very limited abnormal training annotation was sufficient for supervised learning to outperform normal-only training anomaly detection in this task under the fixed labeled-calibration protocol; this is a training-label-efficiency result rather than a claim about the total abnormal annotation required by the full operating pipeline. With all normal training images retained, a ResNet-18 classifier trained with only 1% of abnormal training labels already surpassed PatchCore in AUROC, F1, and FNR; in the finer repeated label-count experiment, the combined AUROC/F1/FNR training-label crossover occurred between 8 and 16 labeled abnormal training images. Thus, for LNG tank cable inspection, normal-only training anomaly detection is useful when abnormal labels are unavailable for model fitting, but a small abnormal training-label budget can quickly justify supervised learning when labeled calibration data are available.

## Figures and Tables

**Table 1:** Duplicate-aware protocol split sizes. Values are shown as normal/abnormal image counts. No duplicate or near-duplicate group crossed train, calibration, or test splits.

Seed	Train	Calibration	Test
11	1486/3228	497/1076	493/1072
22	1485/3228	499/1080	492/1068
33	1492/3230	492/1072	492/1074

**Table 2:** Main image-level benchmark results under duplicate-aware and calibration-controlled evaluation. Values are mean ± standard deviation across three seeds.

Paradigm	Model	AUROC	AUPRC	F1	FNR	Specificity	Accuracy
Normal-only training anomaly	PaDiM-ResNet18	0.8341±0.0851	0.8997±0.0628	0.8389±0.0314	0.1711±0.0837	0.6823±0.1996	0.7827±0.0411
Normal-only training anomaly	PatchCore-ResNet18	0.8909±0.0109	0.9373±0.0117	0.8666±0.0228	0.1519±0.0653	0.7660±0.0732	0.8222±0.0228
Normal-only training anomaly	STFPM-ResNet18	0.7738±0.0157	0.8716±0.0112	0.7224±0.0402	0.3678±0.0630	0.7497±0.0293	0.6692±0.0341
Supervised	ConvNeXt-Tiny	0.9997±0.0005	0.9999±0.0002	0.9959±0.0038	0.0065±0.0059	0.9966±0.0042	0.9945±0.0052
Supervised	EfficientNet-B0	0.9999±0.0001	1.0000±0.0000	0.9956±0.0029	0.0072±0.0044	0.9966±0.0031	0.9940±0.0039
Supervised	ResNet-18	0.9993±0.0004	0.9997±0.0002	0.9936±0.0005	0.0096±0.0005	0.9932±0.0012	0.9913±0.0007

**Table 3:** Abnormal training-label-budget ResNet-18 results. All normal training images were retained; only abnormal labeled training samples used for model fitting were varied. The labeled calibration split was held fixed across budgets. Values are mean ± standard deviation across three seeds. The 100% row uses the same runs as the fully supervised ResNet-18 baseline to avoid a duplicated-condition inconsistency.

Abnormal Training-Label Ratio	Approx. Abnormal Training Labels	AUROC	AUPRC	F1	FNR	Specificity
1%	33	0.9702±0.0036	0.9884±0.0013	0.9408±0.0076	0.0990±0.0185	0.9690±0.0116
5%	162	0.9918±0.0063	0.9967±0.0025	0.9745±0.0124	0.0405±0.0208	0.9791±0.0071
10%	323	0.9959±0.0024	0.9984±0.0009	0.9812±0.0065	0.0312±0.0147	0.9872±0.0072
20%	646	0.9975±0.0019	0.9990±0.0008	0.9828±0.0076	0.0287±0.0136	0.9885±0.0071
50%	1615	0.9990±0.0007	0.9996±0.0003	0.9895±0.0032	0.0186±0.0070	0.9953±0.0031
100%	3229	0.9993±0.0004	0.9997±0.0002	0.9936±0.0005	0.0096±0.0005	0.9932±0.0012

**Table 4:** Fine abnormal training-label-count crossover analysis for ResNet-18. Values are mean ± standard deviation across 15 runs per label count, using three seeds and five subset draws. The labeled calibration split was held fixed across all label counts. PatchCore achieved AUROC 0.8909±0.0109, F1 0.8666±0.0228, and FNR 0.1519±0.0653 in the main calibrated comparison.

Abnormal Training Labels	Runs	AUROC	AUPRC	F1	FNR
4	15	0.8112±0.0464	0.9156±0.0239	0.7575±0.0494	0.3496±0.0661
8	15	0.9040±0.0286	0.9601±0.0137	0.8575±0.0336	0.2186±0.0453
16	15	0.9594±0.0074	0.9840±0.0030	0.9255±0.0106	0.1198±0.0196
24	15	0.9732±0.0075	0.9896±0.0029	0.9430±0.0123	0.0973±0.0236
32	15	0.9789±0.0056	0.9917±0.0021	0.9533±0.0088	0.0784±0.0176
64	15	0.9830±0.0092	0.9934±0.0034	0.9631±0.0112	0.0616±0.0204

## Data Availability

The source images analyzed in this study are from the LNG tank quality inspection image dataset provided by AI-Hub, Republic of Korea (https://www.aihub.or.kr/ (accessed on 26 August 2026)). The source images are subject to AI-Hub access and redistribution terms and are not redistributed with this manuscript. To support research reproducibility without redistributing copyrighted image files, experimental source code, environment specifications, duplicate-audit manifests, train/calibration/test split metadata, frozen thresholds, and evaluation scripts are publicly available in the GitHub repository at https://github.com/Altaire-coder/lng-cable-inspection (accessed on 26 August 2026).

## Abbreviations

The following abbreviations are used in this manuscript:

LNG	Liquefied Natural Gas
STFPM	Student–Teacher Feature Pyramid Matching
FNR	False-Negative Rate
MCC	Matthews Correlation Coefficient
